# Synthesis and Characterization of PtTe_2_ Multi-Crystallite Nanoparticles using Organotellurium Nanocomposites

**DOI:** 10.1038/s41598-017-10239-8

**Published:** 2017-08-29

**Authors:** Javier Fernández-Lodeiro, Benito Rodríguez-Gónzalez, Fernando Novio, Adrián Fernández-Lodeiro, Daniel Ruiz-Molina, José Luis Capelo, Alcindo A. dos Santos, Carlos Lodeiro

**Affiliations:** 1 BIOSCOPE Group, UCIBIO@REQUIMTE, Chemistry Department, Faculty of Science and Technology, University NOVA of Lisbon, Caparica, 2829-516 Portugal; 2 ProteoMass Scientific Society, Madan Parque, Building VI, Office 23, Faculty of Sciences and Technology, Campus de Caparica, 2829-516 Caparica, Portugal; 3 Scientific and Technological Research Assistance Centre (CACTI), University of Vigo, Lagoas-Marcosende, Vigo, Spain; 4 Instituto de Química, Universidade de São Paulo, Av. Prof. Lineu Prestes, 748, CxP.26077, São Paulo, 05508-000 Brazil; 5 Catalan Institute of Nanoscience and Nanotechnology (ICN2), CSIC and The Barcelona Institute of Science and Technology, Campus UAB, Bellaterra, 08193 Barcelona, Spain

## Abstract

Herein, we report the synthesis of new PtTe_2_ multi-crystallite nanoparticles (NPs) in different sizes through an annealing process using new nanostructured Pt-Te organometallic NPs as a *single source precursor*. This precursor was obtained in a single reaction step using Ph_2_Te_2_ and H_2_PtCl_6_ and could be successfully size controlled in the nanoscale range. The resulting organometallic composite precursor could be thermally decomposed in 1,5 pentanediol to yield the new PtTe_2_ multi-crystallite NPs. The final size of the multi-crystallite spheres was successfully controlled by selecting the nanoprecursor size. The sizes of the PtTe_2_ crystallites formed using the large spheres were estimated to be in the range of 2.5–6.5 nm. The results provide information relevant to understanding specific mechanistic aspects related to the synthesis of organometallic nanomaterials and nanocrystals based on platinum and tellurium.

## Introduction

The development of materials at the nanoscale has attracted the attention of the scientific community because of their new and improved properties compared with those of their bulk counterparts^1^. Their physicochemical properties have been revealed to be dependent on their final composition, size and shape, among other factors^2–4^. In this regard, nanostructured metal chalcogenide (MC) materials are the subject of increasing research as a result of the numerous applications reported for these systems, primarily in electronics and energy conversion and storage^3–6^, but also in catalysis^7^, aerogel fabrication^8, 9^ semiconducting materials^10^, among others.

Among the different MCs reported in the literature, binary systems containing tellurium have been extensively explored in recent years, largely due to their potential applications in memory devices^11^, photovoltaic cells^12^, thermoelectrics^13^, catalysis^14^ and biochemical^15^ applications. Specifically, binary-phase systems based on Pt-Te provide an excellent combination of platinum as metal and tellurium as a semiconductor to provide enhanced thermoelectric properties in binary-phased nanocomposites^16^ or photothermal therapy^17^.

A convenient route for the production of MC materials is the use of organometallic derivatives as a *single source precursor* by thermal decomposition^18–23^.

The chemistry of platinum with sulphur derivatives has been widely explored; to a lesser extent, so has that with selenium-containing ligands. By contrast, organic tellurium-based ligands have received less attention, such as in the case of organic ditellurites (R_2_Te_2_). This knowledge gap regarding the role of organic tellurides in coordination chemistry led to early assumptions that they behaviour similar to their chalcogenide counterparts S and Se; however, the rapid development of this field in recent decades has led to this preconception being discarded^24^.

It is commonly accepted that R_2_Te_2_ produces a variety of tellurolate metal complexes with low-valent Pt precursors through different mechanisms, with the most common being Te-Te oxidative addition and/or Te-C reductive cleavage^24^. The evident structural differences between these platinum tellurolate complexes have revealed their large dependence on different experimental factors, such as the type of platinum precursor applied, the nature of the R group in the Te reagent, and the solvent or temperature used during the reaction^24–28^. However, reports about intact coordinated R_2_Te_2_ compounds are limited^29^. Moreover, to the best of our knowledge and in contrast to the commonly used low-valent platinum organometallic precursors, the use of platinum precursors in high oxidation states has been rarely reported.

Recently, our research group reported on the spontaneous tendency of Ph_2_Te_2_ to reduce Au(III) into Au(0) nanoparticles (NPs)^30^. This reaction presumably occurs via Te-Te cleavage with concomitant formation of PhTeCl_3_ and a Au(I) intermediate. This phenyl tellurium trihalide is prone to hydrolysis and could be transformed into oxohalides (PhTe(O)X_n_), tellurinic acids (PhTeOOH) or their corresponding anhydrides [PhTe(O)_n_] as a result of different hydrolysis stages^31, 32^. Additionally, in the presence of O_2_ and H_2_O as well as coordinative solvents, the photodecomposition of Ph_2_Te_2_ produced similar tellurinic acids or anhydride derivatives^33^. These organotellurium derivatives with a simple phenyl group usually evolve into a random polymeric structure via aggregation/condensation processes^34^, producing an organometallic shell over gold cores. With these precedents in mind, we have now applied this novel organotellurium chemical approach to fabricate new monodisperse and size-modulated (nano- and micrometre scale) organometallic Pt-Te NPs, which can be easily dispersed in common organic and environmentally friendly solvents. Moreover, multi-crystallite PtTe_2_ NPs with well-defined spherical shapes and dimensions (depending on the original size of the precursor) were obtained upon annealing the initial Pt-Te polymeric nanomaterial. These results provide an exciting opportunity to advance our knowledge of organotellurium chemistry applied to noble metal nanomaterial fabrication and provide an opportunity to better comprehend the properties of these new platinum/tellurium nanomaterials.

## Results and Discussion

### Synthesis and morphological characterization of organometallic Pt-Te NPs

An acetonitrile solution of Ph_2_Te_2_ and H_2_PtCl_6_ was used as the starting material for the synthesis of Pt-Te NPs. The coordinating nature of this solvent plays a major role during the photodecomposition of the Ph_2_Te_2_ to form phenyltellurinic anhydride derivatives^33^. In a typical synthesis, a boiling acetonitrile solution containing H_2_PtCl_6_ was quickly added to an acetonitrile solution containing Ph_2_Te_2_ (for more details, see the Experimental Section). At the moment of addition, the dark red solution shifted to reddish brown, after which it was left for one hour at boiling temperature, resulting in the formation of a precipitate.

**Figure 1:**
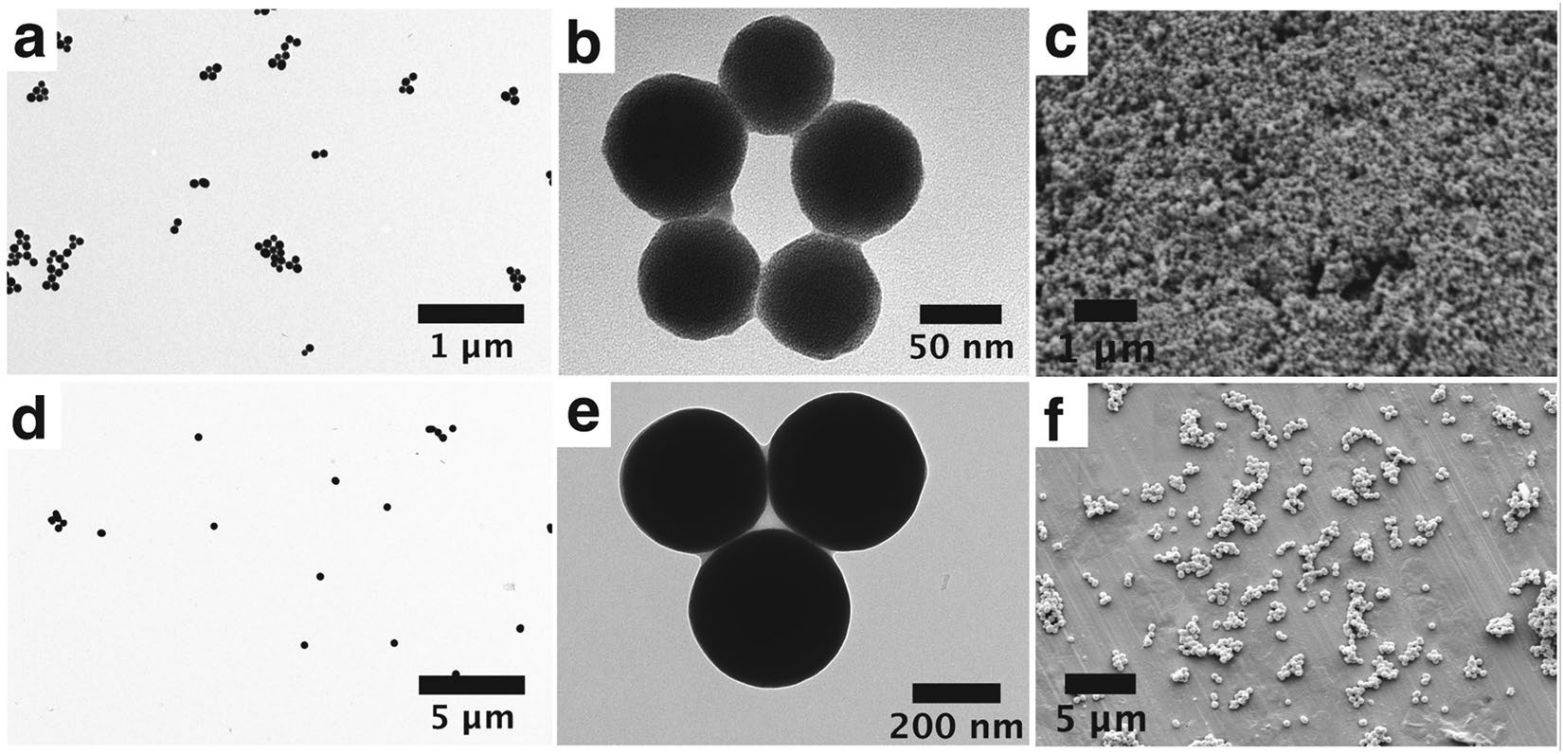
(**a**) Low magnification TEM (**a**,**b**,**d**,**e**) and SEM (**c**,**f**) images of different sizes of organometallic Pt-Te NPs obtained under higher initial reagent concentrations ([Pt(IV)] = 2.10^−4^ M, [Te-Te] = 6.10^−4^ M) (**a**,**b**,**c**) and ([Pt(IV)] = 8.10^−3^ M, [Te–Te] = 2.4.10^−2^ M) (**d**,**e**,**f**).

We also found a correlation between the water percentage during the reaction and the resulting organometallic nanocomposite; a distinctly lower yield and higher polydispersity were obtained at lower water contents. (Figure S1). This result can be correlated with the hydrolysis of phenyl tellurinyl chlorides (PhTeCl_n_) formed as a sub-product of Ph_2_Te_2_ oxidation, similar to that observed for the previously reported gold system^30^. By contrast, no significant differences were observed for the reactions conducted under dark or inert atmosphere conditions (Figures S2 to S5). In this respect, Ph_2_Te_2_ photodecomposition processes or additional oxidation by O_2_ did not show any impact on the final nanostructuring of the obtained polymeric material. Additionally, an increase in the initial reagent concentrations induced a size increase of the final nanoparticles, although with a higher resultant polydispersity (Figures 1 and S6 to S8).

Modifying the reactant sequence addition also modified the reaction output, resulting in a more polydispersed material and a partial loss of the spherical morphology (Figure S9).

The final organometallic Pt-Te NPs can be dispersed in absolute ethanol or water (Figure S10). This observed behaviour with nucleophilic solvents is contrary to that observed with pure condensed tellurinic acid or anhydride derivatives formed in the previous reported gold system^30^, indicating that in this case, the Pt ion was likely directly coordinated into the organometallic structures. This new polymeric material is not dissolved or disrupted in absolute ethanol as previously observed with the gold nanomaterial.

### Chemical characterization of organometallic Pt-Te NPs

Based on the electron microscopy evidence, the materials obtained in the presence or absence of oxygen and/or light have similar nanostructures; thus, we selected samples obtained in the presence of oxygen and light to perform a more complete characterization.

**Figure 2:**
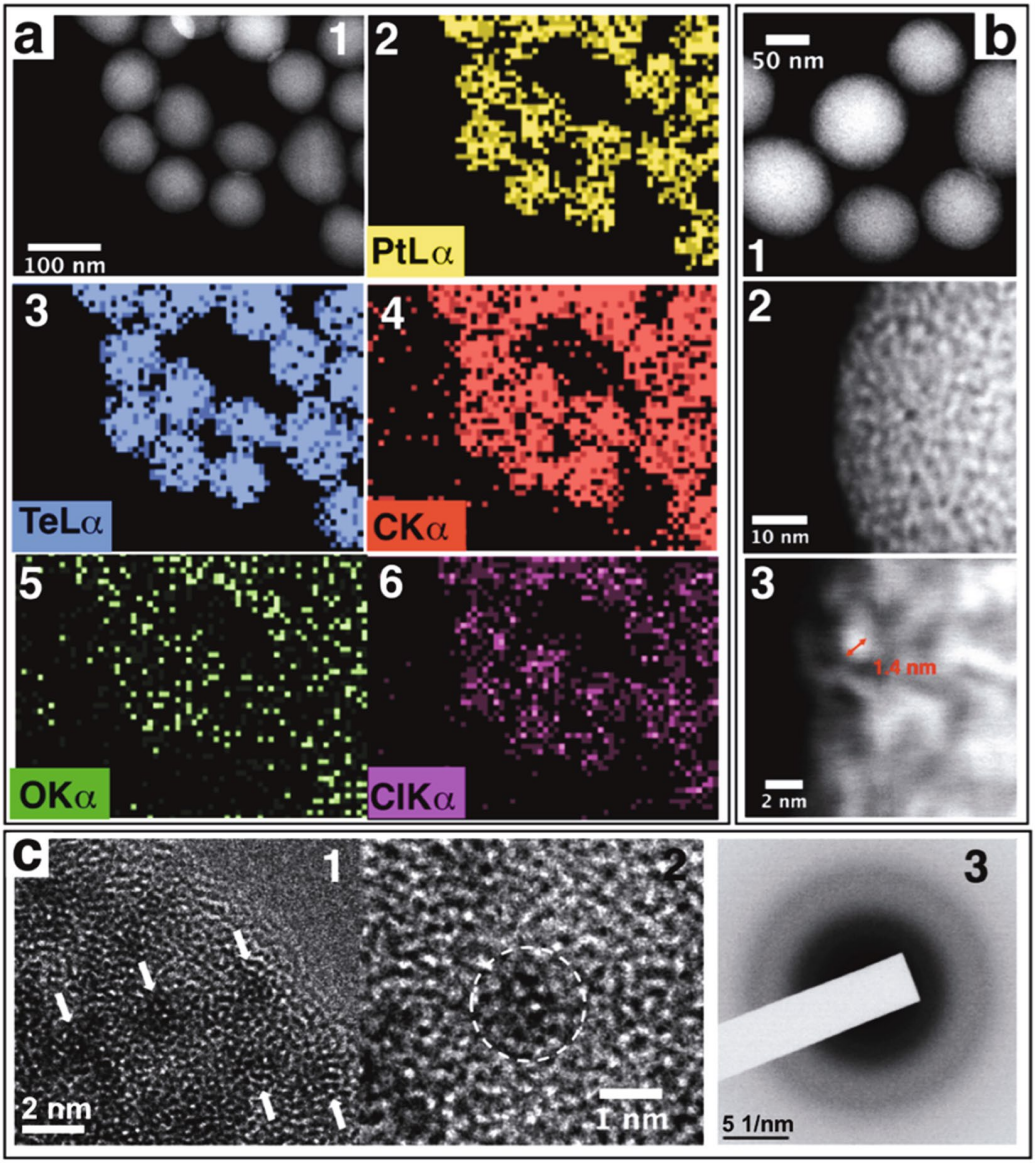
(**a**) STEM-HAADF image of a group of Pt-Te NPs and Te, Pt, C, O and Cl EDS elemental maps (1–6). (**b**) HAADF-STEM image: one group of isolated Pt-Te NPs (1) and two close ups (2) showing the complex structure and the nodules composed of high atomic-number elements (3). (**c**) HRTEM image and diffraction pattern showing the structure and demonstrating the lack of a crystalline structure in the nodules.

Figure 2a (panels 1 to 6) shows a high-angle annular dark field (HAADF) image together with five energy-dispersive X-ray elemental maps (EDS) obtained for a group of Pt-Te NPs. These maps show that the major elements present in the particles are tellurium, platinum and carbon. In addition, the presence of oxygen and chlorine as minor elements was also confirmed. Similar results were obtained with the EDS X-ray microanalysis obtained from an individual particle (Figure S11).

**Figure 3:**
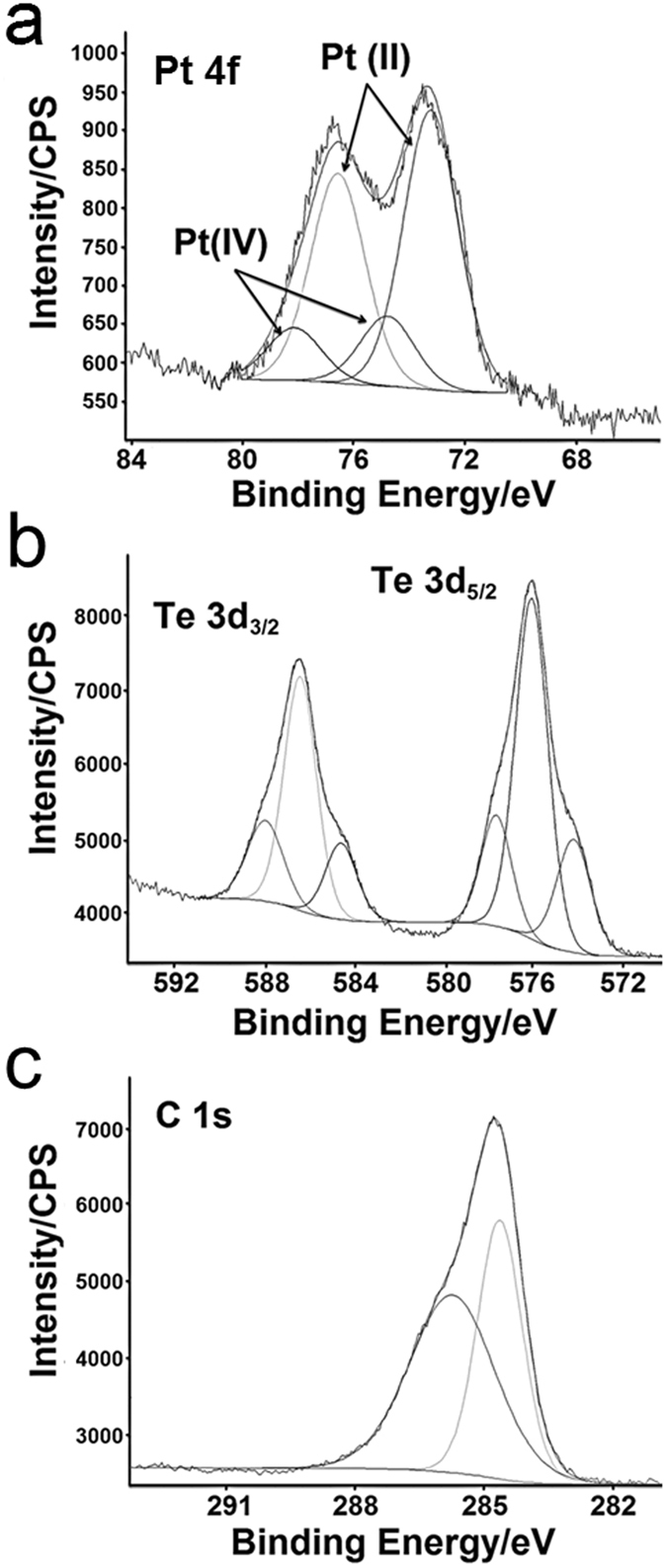
XPS spectrum of organometallic Pt-Te NPs. Binding energy spectrum of Pt 4 f (**a**), Te 3d_3/2_ and 3d_5/2_ (**b**), and C 1 s (**c**).

Even though the spectrum shows a high Cu peak due to the contribution of the copper grid used in the transmission electron microscope (TEM) sample preparation, the main elements present in the particle are Te, Pt, C, O and Cl. Combined high-resolution TEM (HRTEM) and HAADF images were also obtained, as shown in Fig. 2b 2,3. As seen in Fig. 2b 1, the general trend indicates an increase in brightness from the outside to the centre of the particles, consistent with a spherical geometry and a mass-thickness contrast mechanism. The presence of a hole, or a non-uniform distribution of the elements within the particle, was discarded in light of the elemental profiles along the diameter of the particle (Figure S12). Images at higher magnification (Fig. 2b 2,3) showed the presence of a complex inner nanostructure; the contrast was not distributed evenly but showed grainy small nodules forming with higher contrast levels. These images suggest that Te and/or Pt tend to concentrate in these approximately 1.4 nm nodules, and the other elements (C, O, and Cl) are mainly surrounding those nodules. Interestingly, TEM images at higher magnification (Fig. 2c) did not show the presence of lattice fringes in areas containing these nodules, nor any feature indicating the presence of a crystalline structure in the centre of the nodules. The amorphous character of the obtained Pt-Te NPs was confirmed by the electron diffraction pattern obtained from a group of those particles (Fig. 2c–3) and corroborated by the powder X-ray diffraction pattern obtained for the material (Figure S13). Additionally, characterization using electron energy loss spectroscopy (EELS) is displayed in (Figure S14). The EEL spectrum in the carbon K-edge region shows two bands corresponding to two different electronic bonding states, one assigned to the π C=C bond peak at 285 eV and the other assigned to the σ C-C bond peak close to 297 eV. The oxygen and tellurium edges region is shown in Figure S14b; in this region, the oxygen edge displays a relatively low intensity, especially compared with the intensity and shape of the TeO_2_ edges^35^. This result provides clear evidence of a large oxygen deficit compared with the relative content of oxygen in the TeO_2_. Conversely, the Te edge shows an intense broad band, suggesting the presence of a large Te content and a mixture of electronic states in the Te bonds.

The X-ray photoelectron spectroscopy (XPS) spectrum shown in Figs 3 and S5 shows a high Te (53%) percentage compared with those of C (35%), Pt (7%) and Cl (4.5%). Moreover, two signals can be assessed for Pt, namely, Pt 4f_7/2_ at 73.23 eV, Pt 4f_5/2_ at 76.58 eV and Pt 4f_7/2_ at 74.80, Pt 4f_5/2_78.15 eV. Thus, the nanostructure contains mainly Pt(II) (Pt(II) 4f_7/2_ = 73.23 eV, 4f_5/2_ = 76.58 eV) with a small proportion of Pt(IV) (Pt(IV) 4f_7/2_ = 74.80 eV, 4f_5/2_ = 78.15 eV), and the ratio of the areas of Pt(II):Pt(IV) is 4:1 (see Fig. 3a).

Three components were found for Te, presumably associated with the different oxidation states as revealed by EELS analysis (Fig. 3a). The XPS chemical shifts for tellurium (3d_5/2_ and 3d_3/2_) in different oxidation states appear in a narrow region. The main peaks appear at 576.1 (3d_5/2_) and 586.4 (3d_3/2_) eV, which correspond to telluroxide (TeO) functionalities^36, 37^. If we focus on the 3d_5/2_ sub-spectrum of which one of the minor components (21%) at 574.2 (3d_5/2_) and 584.6 (3d_3/2_) eV could be attributed to (Ph_2_Te_2_)–Pt units, the other minor component (18%) at 577.7 (3d_5/2_) and 588.0 (3d_3/2_) eV should be attributed to oxidized species of telluride. Finally, two energy peaks appear in the C 1 s region associated with aromatic C-C bonds (284.6 eV) and the C-Te bond (285.7 eV) (Fig. 3).

FT-IR spectroscopy of the Pt-Te NPs (Figure S15) show characteristic bands of phenyl C_sp2_-H (3051 cm^−1^), C=C stretches (1568 cm^−1^, 1469 cm^−1^, 1431 cm^−1^), and C–H bending in-plane (1053 cm^−1^, 1014 cm^−1^) and out-of-plane (727 cm^−1^, 686 cm^−1^). Additionally, aromatic ring overtones can be observed in the region of 2000–1630 cm^−1^ ^38, 39^.

Attempts to characterize the nanomaterial with Raman spectroscopy were unsuccessful, as the nanocomposite was burned during the measurements by the action of the laser, and the results were inconclusive.

The composition of the material was analysed by elemental analysis and inductively coupled plasma (ICP). We analysed two different particle sizes, denoted as R4 and R5. In this way, we obtained the following values, expressed as a percentage (R4/R5): %C (22.51/22.81), %H (1.52/1.59), %Pt (26.03/24.37) and %Te (44.55/45.84). The slight difference in percentages obtained may be related to the partial change in the reaction conditions (see Table 1).

**Table 1:** Experimental reaction conditions ([Reagent] in n/L and %H_2_O in V/V).

Sample	Media	[Pt^4+^]_final_	[Te-Te]_final_	%H_2_O	O_2_	Light
R1	CH_3_CN	2 × 10^−4^	6 × 10^−4^	0	P	P
R2	CH_3_CN	2 × 10^−4^	6 × 10^−4^	0.1	P	P
R3	CH_3_CN	2 × 10^−4^	6 × 10^−4^	0.2	P	P
R2.1	CH_3_CN	2 × 10^−4^	6 × 10^−4^	0.1	A	P
R2.2	CH_3_CN	2 × 10^−4^	6 × 10^−4^	0.1	P	A
R4	CH_3_CN	2 × 10^−3^	6 × 10^−3^	0.2	P	P
R5	CH_3_CN/CH_2_Cl_2_	8. × 10^−3^	2.4 × 10^−2^	0.2	P	P

The composition differences obtained by XPS and ICP/AE show increased tellurium and carbon percentages for XPS analysis. The XPS spectrum gives information about the elemental composition of the shallow surface region. As photons possess a limited penetrating energy (up to 10 nm), only those electrons pertaining to atoms near the surface can be counted. This quantitative technique provides the average composition over an approximate 10 nm depth inside the nanoparticle. Taking into account these considerations inherent to the technique, the comparatively increased tellurium and carbon percentages can be explained as a result of the adsorption and/or coordination of the remaining Ph_2_Te_2_ molecules on the surface-formed nanocomposite.

Using thermogravimetric analysis, we determined the thermal behaviour of organometallic Pt-Te NPs with the aim of delimiting the parameters for the annealing process. We observed that the decomposition occurred under multistage process between 25 °C and 1500 °C (Figure S16). The first mass loss (−37.22% completed above 400 °C) is associated with three exothermic changes, as seen in the differential thermogravimetry (DTG) curve (Figure S16), with the first exothermic signal appearing near 200 °C. We believe that this first exothermic signal could be related to the crystallization of PtTe_2_.

### Metal-ligand interaction during the reaction

Metal-ligand interaction and the formation of the Pt-Te NPs was investigated using UV/Vis spectroscopy and FT-ICR-MS (Fourier transform ion cyclotron resonance mass spectrometry) studies.

The spectroscopy profile of Ph_2_Te_2_ presents two absorption bands, one near 300 nm, which was assigned to the nTe-π* transition (phenyl group charge transfer band), and one near 397 nm, corresponding to the nTe-σ* transition (Te-Te charge transfer band)^40, 41^. To study the interaction of the Te-Te residue with the Pt cation, we selected two ligand:metal stoichiometries (L:M = 3:1 and 1:1). In both cases, we observed a time-dependent Te–Te charge transfer band increase upon addition of the metal cation, indicating the coordination interaction between the Pt ions and the Ph_2_Te_2_ ligand (Figure S17).

Surprisingly, FT-ICR-MS analysis, performed just 1 minute after mixing the reagents, showed only the presence of some signals with clear Te isotopic distribution. Interestingly, we observed that the two most intense signals consistent with the Te isotopic distribution (at 616.833 and 853.398 *m/z*) are coincident with those observed in our previous reported studies related to Au-Te nanoparticles (Figure S18)^30^. These results suggest that similar organotellurium derivatives are formed in both systems. Additionally, a signal was observed at 1118.674* m/z* displaying a Te isotopic pattern. This signal was attributed to the empirical formula [C_24_H_21_Cl_2_O_2_Te_4_Pt]^+^, arising from the coordination of a Pt(II) cation with a Ph_2_Te_2_ molecule and a phenyl tellurium oxidized (PhTeO)_2_ derivative resulting from the oxidation of Ph_2_Te_2_, along with two chlorine atoms remaining coordinated to the Pt atom (Figure S19). After 20 minutes of reaction, the mass spectrum signal intensities were drastically reduced. The limited literature related to this type of reaction, associated with the very high kinetics of the involved transformations, hinders a better interpretation of the mass spectrometry analysis, as only some plausible Te- and/or Pt-containing isotopic patterns were detected. Moreover, the low solubility of the formed organometallic oligomers could be attributed to the signal decrease observed during the ESI ionization process.

Comparing the relative percentages of the structure proposed by theoretical FT-ICR-MS calculations with the experimental data, we assume that the final structure is not composed of pure units of [C_24_H_20_Cl_2_O_2_Te_4_Pt]^+^; this “structure-defect” may be attributed to a subsequent evolution of the proposed structure into oligomers/polymers.

Based on our previous reports^30^ and others^37, 42^ related to the ability of organic ditellurides to reduce Au(III) to Au(I), we propose here that Ph_2_Te_2_ should act as a Lewis base, reducing Pt(IV) to Pt(II) as was observed by XPS analysis. Consequently, oxidized phenyl tellurinyl chlorides should be formed. These halogenated tellurium derivatives are known to undergo different hydrolysis reactions under specific conditions, producing oxohalides (PhTe(O)X_n_), tellurinic acids (PhTeOOH) or anhydride [(PhTeO)_n_] derivatives^31, 32^. These oxygenated tellurium entities probably originate from the first obtained tellurium chloride species, which imputes high electrophilic character to the tellurium atom, being consequently more susceptible to react with water, for instance.

This hypothesis is linked with the low yield obtained at lower water contents, under which hydrolytic events are drastically reduced, hampering the progress of the nanoparticle formation. The low solubility of the organometallic structure formed in acetonitrile favours particle formation.

### Synthesis and characterization of PtTe_2_ multi-crystallite NPs

As discussed, when the organometallic Pt-Te NPs were subjected to thermogravimetric analysis, an exothermic signal was observed at approximately 200 °C, which was believed to correspond to the crystallization process of a Pt-Te species. Thus, we decided to cover the nanoparticles with a stabilizing polymer, selecting PVP for this purpose. Examining high boiling point solvents (above 200 °C) in which the nanoparticles as well as the PVP could be solubilized, 1,5-pentanediol was found to be efficient for the annealing process. The thermal decomposition was completed at 220 °C in 1 h. The red/brown solution turned black, indicating the formation of metallic PtTe_2_ nanoparticles.

The final size of these multi-crystallite metallic NPs was highly dependent on the organometallic precursor. As an example, annealing R1 (approximately 80 ± 20 nm) produced multi-crystallite metallic nanoparticles of approximately 45 ± 15 nm (Figure S20). The same effect was observed for R5 (Figure S21). We strongly believe that the pre-adsorbed PVP polymer should prevent the aggregation of bulk PtTe_2_ material during the thermal decomposition, thereby guaranteeing the final spherical shape of the PtTe_2_ multi-crystallite NPs.

**Figure 4:**
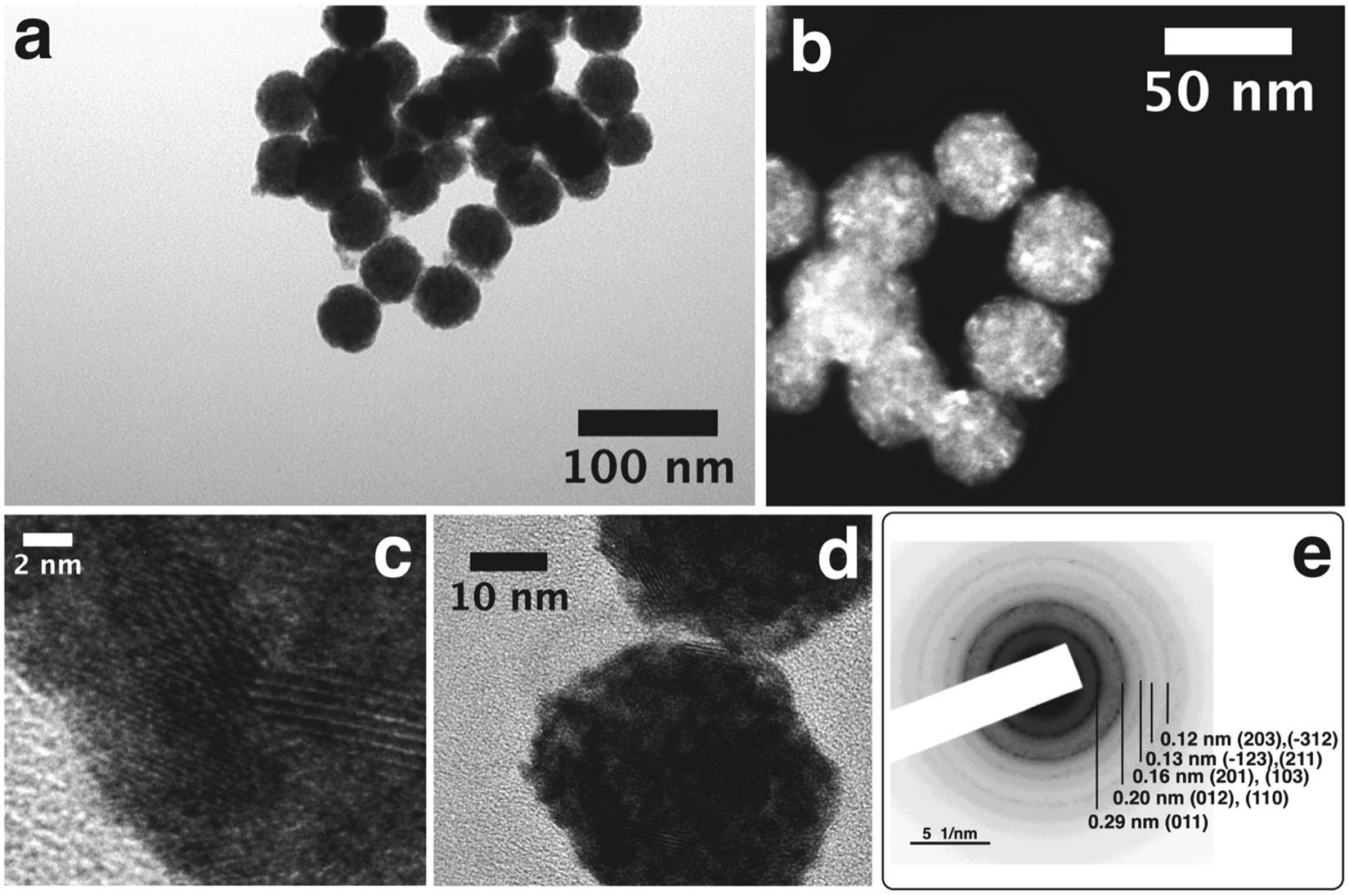
TEM (**a**), and STEM (**b**) images of PtTe_2_ multi-crystallite NPs obtained after the annealing process, HRTEM images of isolated NPs showing lattice image fringes (**c**,**d**) and electron diffraction pattern obtained from a group of PtTe_2_ multi-crystallite NPs showing clear diffraction rings; this pattern was indexed on the basis of the PtTe_2_ crystalline structure (P −3 m 1, SG: 164) (**e**).

TEM and HRTEM micrographs are shown in Fig. 4. Interestingly, these particles show clear lattice fringes, which reveals the crystalline nature of these nanoparticles.

Their crystalline phase was clearly determined by indexing the electron diffraction pattern shown in Fig. 4e; this pattern matches quite well with the PtTe_2_ crystalline structure obtained from the database^43^. At this point, we firmly believe the annealed nanoparticles were formed of a PtTe_2_ (P −3 m 1, SG: 164) crystalline structure. The annealing crystallization stage is characterized by two new features: first, a large drop in the intensity of the C-K_α1–2_ intensity shown in Figure S22, and second, the presence of brighter areas in the STEM image showing crystalline contrast, contrary to the case of the Pt-Te organometallic polymeric particles (Fig. 4b). These two features demonstrate the low carbon content of the PtTe_2_ nanoparticles and indicate the presence of small crystallites in the spheres sized between 2.5–6.5 nm (Figure S23). Similar results were obtained when annealing the R5 sample (Figure S24).

These results clearly demonstrate that the annealing process transformed the initial amorphous organometallic Pt-Te nanocomposite into well-formed, spherical PtTe_2_ multi-crystallite NPs.

## Methods

### Synthesis of organometallic Pt-Te NPs

The one-pot synthesis of the nanoparticles was performed as follows: an acetonitrile (2 mL) solution of Ph_2_Te_2_ (3 × 10^−5^ mol) was quickly added into a two-neck round bottom flask, coupled with a drying tube, containing H_2_PtCl_6_.H_2_O (1 × 10^−5^ mol) in boiling acetonitrile (48 mL). The resulting dark red solution was maintained under reflux for an additional 1 h and then cooled on an ice/water bath. Immediately after cooling, the nanoparticles were isolated by centrifugation (8000 rpm × 30 min). The centrifugation procedure was repeated three times. To study the effects of water, light and/or oxygen on the formation of the organometallic Pt-Te NPs, this general procedure was performed in different conditions (summarized in Table S1). The syntheses in the presence of oxygen were performed under open atmosphere. For the synthesis in the absence of oxygen, the entire reaction occurred under nitrogen atmosphere, and the solvent was deoxygenated under US and nitrogen bubbling for 30 minutes prior to use.

### Reverse order of reagent addition (entry R4)

A solution of 2 mL of acetonitrile containing 1 × 10^−4^ mol of H_2_PtCl_4_ was added to 48 mL of boiled acetonitrile solution containing 3 × 10^−4^ mol of Ph_2_Te_2_ Table 1.

### Synthesis in the presence of CH_2_Cl_2_/acetonitrile mixture (entry R5)

A dichloromethane/acetonitrile (1:1, 1 mL) solution of Ph_2_Te_2_ (3.6 × 10^−4^ mol) was quickly added to a flask containing a boiling acetonitrile (14 mL) solution of H_2_PtCl_6_ (1.2 × 10^−4^ mol). The solids were isolated by filtration on a sintered glass plate (No. 4) and washed repeatedly with acetonitrile and diethyl ether. The solid was then dispersed in acetonitrile (50 mL) under sonication and then isolated by centrifugation (3 cycles of 2000 rpm × 10 min) Table 1.

### Synthesis of multi-crystallite NPs

The synthesis of PtTe_2_ multi-crystallite NPs used an annealing process, which consisted of dissolving the organometallic Pt-Te NPs (11 mg) in 1,5-pentanediol (10 mL) under sonication. PVP40 (50 mg) was added to this brownish-red solution, and the sonication was maintained for an additional 10 minutes. The mixture was heated to 220 °C during 1 h. Colour changes from brownish-red to black developed during the heating process. After this heating time, the reaction mixture was cooled in an ice/water bath, and then the resulting crystalline nanoparticles were isolated by centrifugation 2 cycles (10000 rpm × 1 h) followed by washing with ethanol, ending the process by resuspending the clean PtTe_2_ multi-crystallite NPs in absolute ethanol.

### Fourier transform ion cyclotron resonance mass spectrometry (FT-ICR MS) analysis

FT-ICR MS studies were performed at the Scientific and Technological Research Assistance Centre (CACTI), University of Vigo using an APEXQe FT-ICR MS (Bruker Daltonics, Billerica, MA, USA), equipped with a 7 T actively shielded magnet. To follow the reaction by FT-ICR MS, 100 μL aliquots were removed directly from the reaction medium at different time intervals (1, 20 and 60 minutes). Each aliquot was diluted to 1 mL with 70:29.9:0.1 (v/v/v) CH_3_CN/water/formic acid prior to injection into the mass spectrometer.

Ions were generated using a Combi MALDI-electrospray ionization (ESI) source. The mass spectra were obtained by ionization via an electrospray, using a voltage of 4500 V applied to the needle and a counter voltage of 300 V applied to the capillary.

### Transmission electron microscopy (TEM) analysis

Microscopy analyses were performed at the CACTI, University of Vigo. A JEOL JEM1010 TEM working at 100 kV was used to obtain low-magnification TEM images. A JEOL JEM 2010F field-emission gun TEM working at 200 kV was used to obtain HRTEM images. EDS maps were acquired by coupling the scanning unit of the microscope to an INCA 200 EDS system. EEL spectra were collected in STEM mode using a Gatan GIF Quantum spectrometer with an energy resolution of 1.75 eV (FWHM Zero Loss peak), 0.5 eV/channel energy dispersion and an EELS collection semi-angle of 16 mrad. The EEL spectral background was subtracted using standard DigitalMicrograph routines. All TEM samples were prepared by placing drop of the sample on a TEM copper grid coated with holey carbon thin film and then air dried. To avoid the interference due to the carbon foil grid, the EEL spectra were collected from areas of sample situated in a hole.

### Scanning electron microscopy (SEM)

SEM images were taken using a Quanta environmental scanning electron microscope (FEI Quanta 650) operating between 20 and 5 kV with a spot size = 3.5. Samples dispersed in ethanol were deposited in aluminium holders used as support and metallized with platinum sputtering (sputter coating = 3 nm) before analysis.

### X-ray photoelectron spectroscopy (XPS)

XPS measurements were performed using a Phoibos 150 analyser (SPECS GmbH, Berlin, Germany) in ultra-high vacuum conditions (base pressure 1 × 10^−10^ mbar) with a monochromatic aluminium Kα X-ray source (1486.74 eV). All the spectra were referenced to aliphatic carbon at a binding energy of 284.8 eV.

### UV/Vis and FT-IR spectroscopy studies

The UV/Vis spectroscopy studies were performed using a JASCO 650 spectrophotometer provided by the PROTEOMASS-BIOSCOPE facility. A Bruker TENSOR (REQUIMTE-Chemistry Department, FCT-UNL) spectrophotometer was used to obtain the FT-IR spectra; All FT-IR experiments were performed in KBr disks.

### Inductively coupled plasma (ICP) analysis

The Pt and Te contents in each studied sample were determined in the REQUIMTE-Chemistry Department, FCT-UNL analytical laboratory using an ICP instrument from Horiba Jobin–Yvon (France, model Ultima), equipped with an RF of 40.68 MHz, a 1.00 m Czerny–Turner monochromator (sequential), and an AS500 autosampler.

### Elemental analysis

The elemental analysis was done in the REQUIMTE-Chemistry Department, FCT-UNL analytical laboratory by using an Elementar Thermo Finnigan-CE Instruments (Italy) Flash EA 1112 CHNS series.

### Thermogravimetric analysis

The thermogravimetric analysis was performed in the CACTI, University of Vigo using a Setsys evolution (TG/DSC/DTA) Setaram instrument.

## Conclusion

Organotellurium chemistry was applied successfully to the construction of new platinum nanomaterials; this approach provides a versatile chemical tool in the assembly of novel nanostructured materials. The redox and hydrolytic properties shown by the organic tellurium-based entities were necessary for their role in the construction and stabilization processes. The spontaneity of the Pt cation reduction promoted by the starting ditelluride reagent, associated with the easily adjustable experimental conditions to promote transformations based on the tellurium moiety, can be considered the key draw of this new synthetic strategy for obtaining well-defined nanoparticles.

New organometallic Pt-Te NPs were formed by a mixture of Te, Pt and C, plus minor amounts of O and Cl. This inhomogeneous mixture of elements produces Pt and Te nodules, conferring the final spherical shape to the particles. Each of the nodules is approximately 1.4 nm in diameter. Surrounding all the Pt-Te nodules are lighter elements, mainly C and O, acting as agglutinant agents. The interior of the nodules appear to be a mixture of organometallic Pt compounds, not crystalline Pt. EDS analysis demonstrates a low carbon content in the PtTe_2_ multi-crystallite NPs relative to the initial composite NPs. The size of the PtTe_2_ crystallites forming the large spheres was estimated to range from 2.5 to 6.5 nm. Further studies related to applications of these new nanoparticles are in progress.

## Electronic supplementary material

Supplementary Information 
