# Supplementary material for: Synthesis and Characterization of PtTe2 Multi-Crystallite Nanoparticles using Organotellurium Nanocomposites
Source: Sci Rep. 2017 Aug 29;7:9889. doi: 10.1038/s41598-017-10239-8 (PMC5575282; doi:10.1038/s41598-017-10239-8)
Supplement: Supplementary file 1 — Supplementary Information [file 41598_2017_10239_MOESM1_ESM.pdf]

Synthesis and Characterization of $PtTe_2$ Multi-Crystallite Nanoparticles using Organotellurium Nanocomposites.

Javier Fernández-Lodeiro,[a,b,c] Benito Rodríguez-Gonzalez,[d] Fernando Novio,[e] Adrián Fernández-Lodeiro,[a,b] Daniel Ruiz-Molina,[e] José Luis Capelo,[a,b] Alcindo A. dos Santos,[c] Carlos Lodeiro*[a,b]*

[a] BIOSCOPE Group, LAQV@REQUIMTE, Chemistry Department, Faculty of Science and Technology, University NOVA of Lisbon, Caparica, 2829-516,Portugal.

[b] ProteoMass Scientific Society, Madan Parque, Building VI, Office 23, Faculty of Sciences and Technology, Campus de Caparica, 2829-516. Caparica. Portugal.

[c] Scientific and Technological Research Assistance Centre (CACTI), University of Vigo, Lagoas-Marcosende, Vigo, Spain

[d] Instituto de Química, Universidade de São Paulo, Av. Prof. Lineu Prestes, 748, CxP.26077, São Paulo 05508-000, Brazil.

[e] Catalan Institute of Nanoscience and Nanotechnology (ICN2), CSIC and The Barcelona Institute of Science and Technology, Campus UAB, Bellaterra, 08193 Barcelona, Spain

*Corresponding authors: Javier Fernández-Lodeiro (j.lodeiro@fct.unl.pt) / Carlos Lodeiro (cle@fct.unl.pt)

1. Materials

Hydrogen hexachloroplatinate (IV) hydrate (99.99 % metal basis, CAS: 26023-84-7), Diphenyl ditelluride (98%, CAS: 32294-60-3), Calcium hydride (95%, CAS: 7789-78-8), Polyvinylpyrrolidone (PVP40, CAS: 9003-39-8), 1,5-Pentanediol (97%, CAS: 111-29-5) were purchased from Sigma Aldrich.

2. Additional characterizations

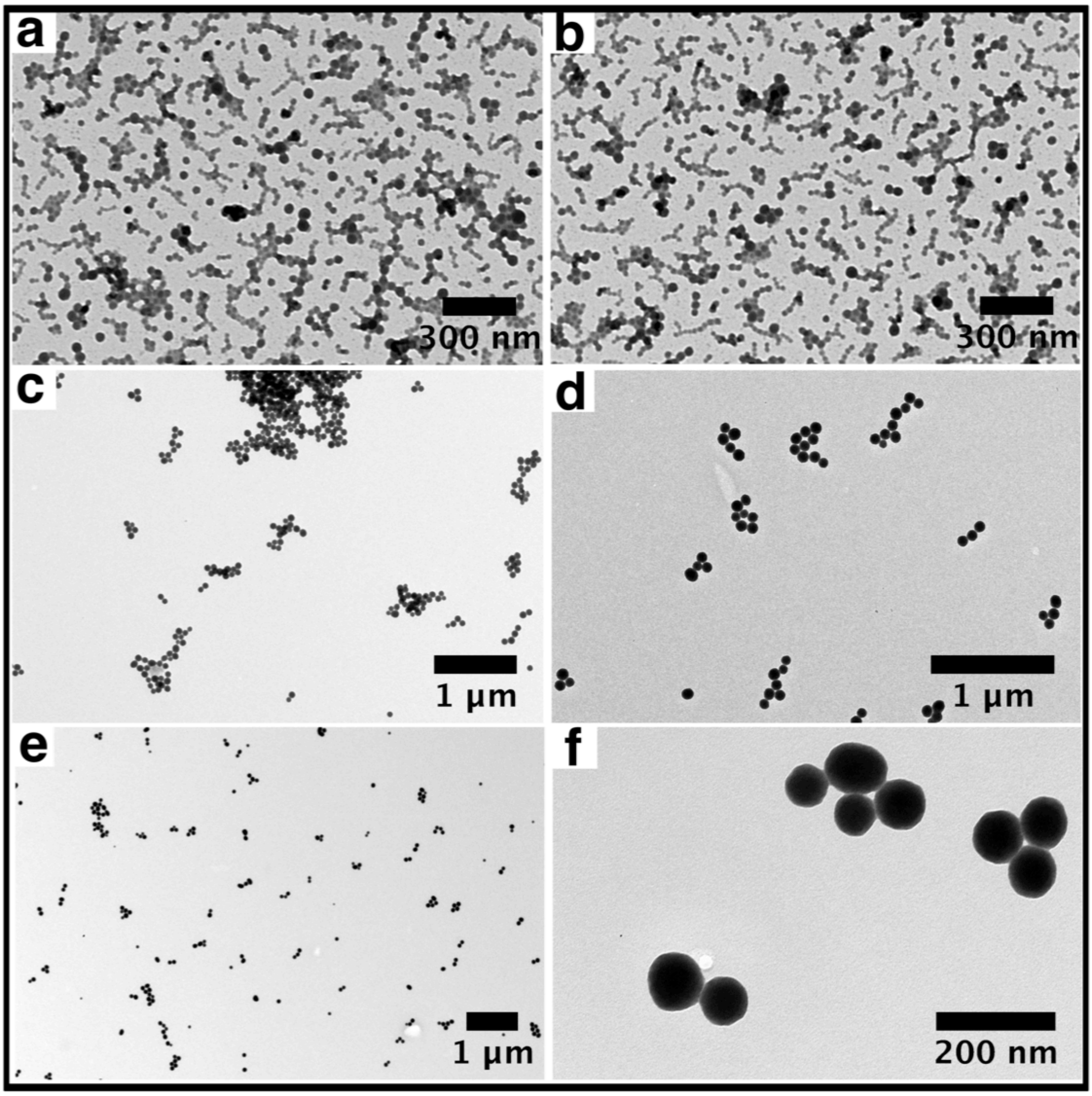

Figure S1. Low magnification electron transmission microscopy images obtained for **R1** (a, b), **R2** (c, d) and **R3** (e, f).

Under low water-content conditions the crude reaction present red/Brown colour with transparent aspect. Only with centrifugation process at 14000x1h was possible to obtain enough material for characterization. **R2** and **R3** were obtain in pure form with three centrifugation cycle at 8000 rpmx30 min. in acetonitrile.

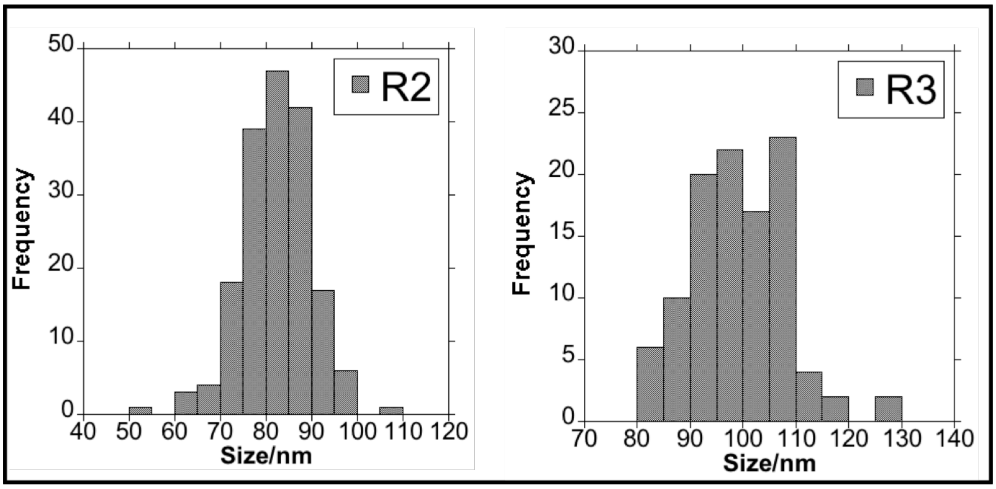

Figure S2: Histogram obtained for **R2** and **R3** samples. (Histogram was obtained counting a minimum of 100 particles per sample).

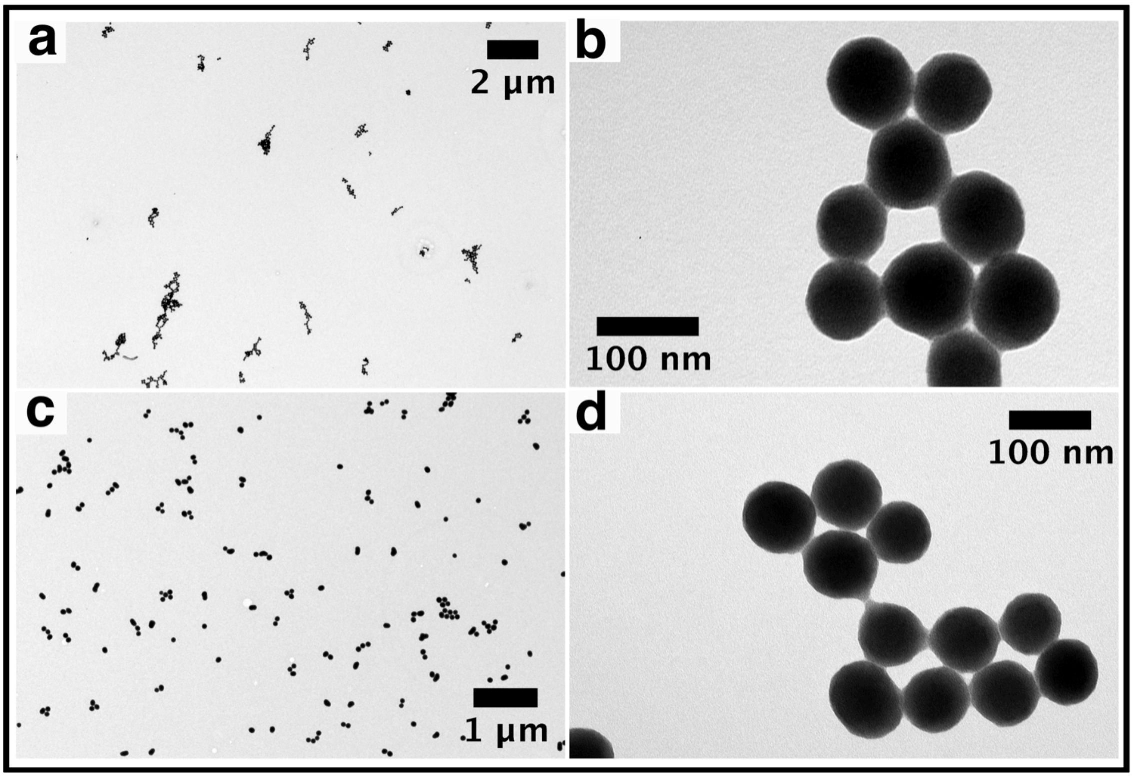

Figure S3: Low magnification TEM images obtained for **R2.1** (**a** and **b**), **R2.2** (**c** and **d**).

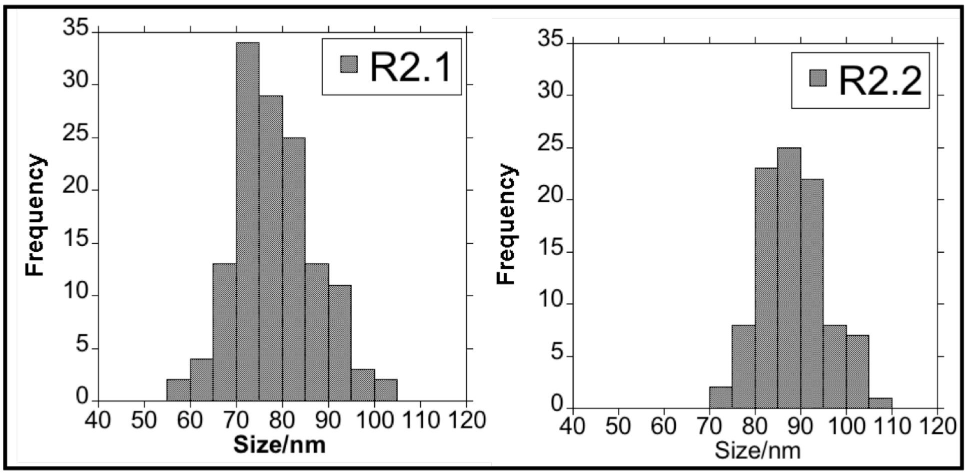

Figure S4: Histogram obtained for **R2.1** and **R2.2** samples.

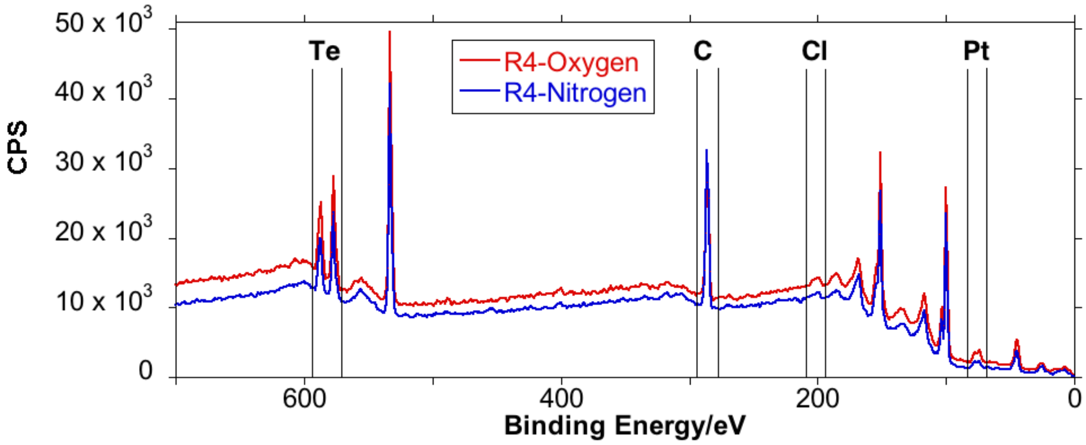

Figure S5: Overview X-Ray photoelectron spectroscopy of **R4** obtaining under N_2 or O_2 atmosphere.

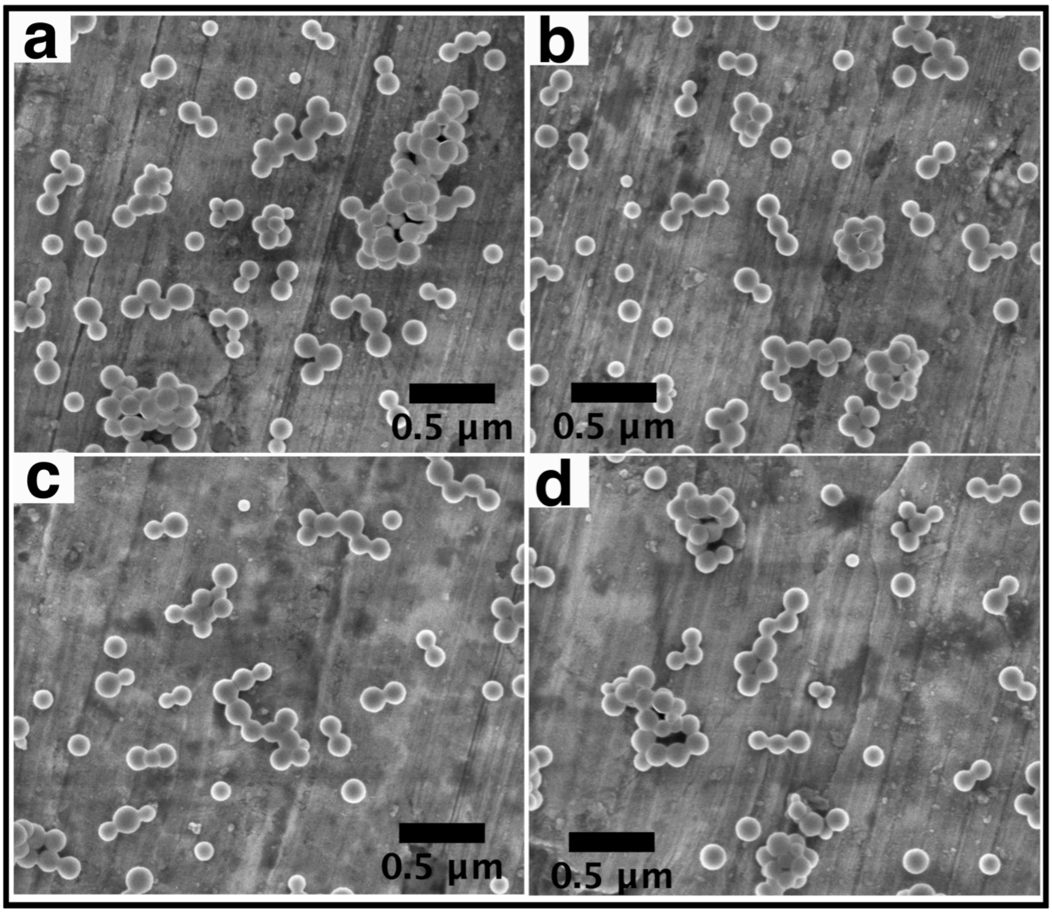

Figure S6: Low magnification SEM images obtained for **R4**

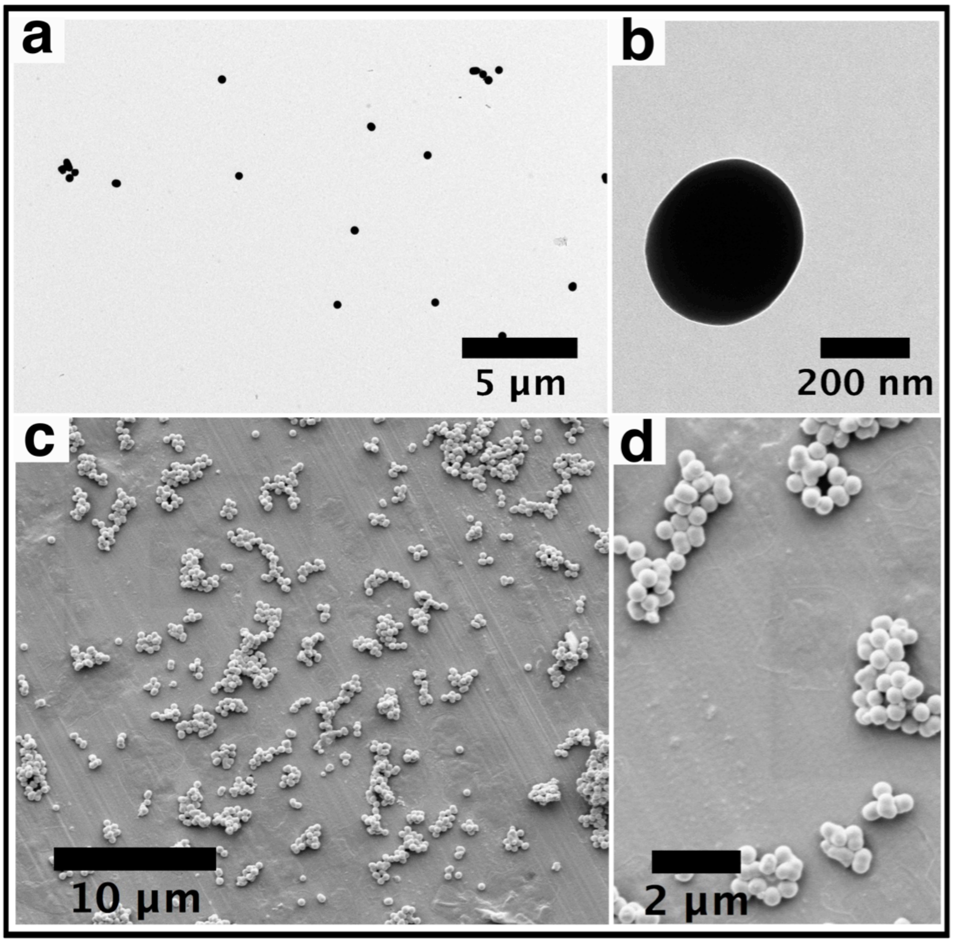

Figure S7: Low magnification TEM (**a**, **b**) and SEM (**c**, **d**) images obtained for **R5.**

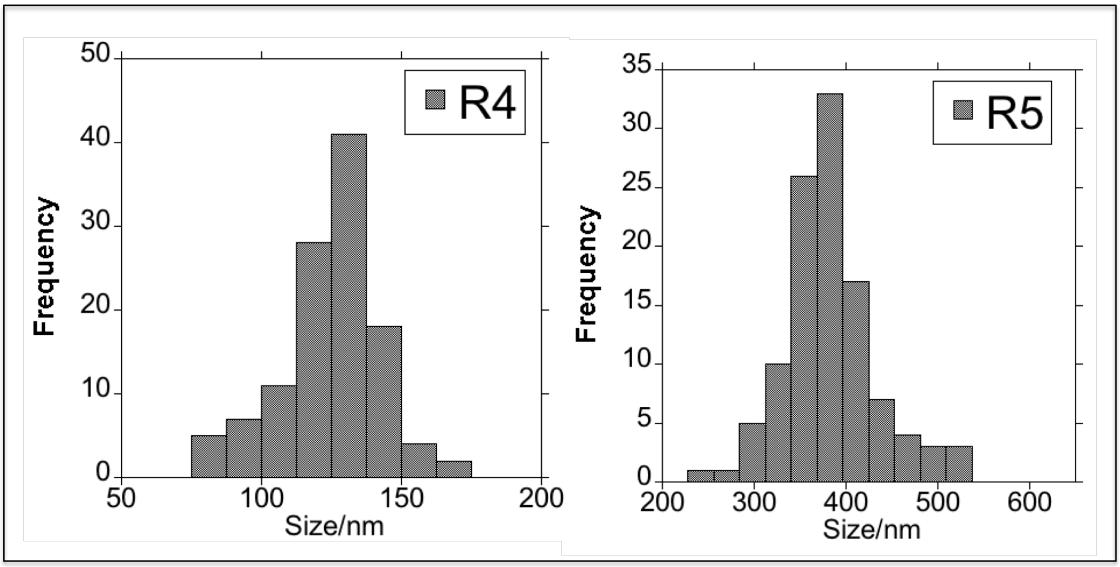

Figure S8: Histogram obtained for **R4** and **R5** samples.

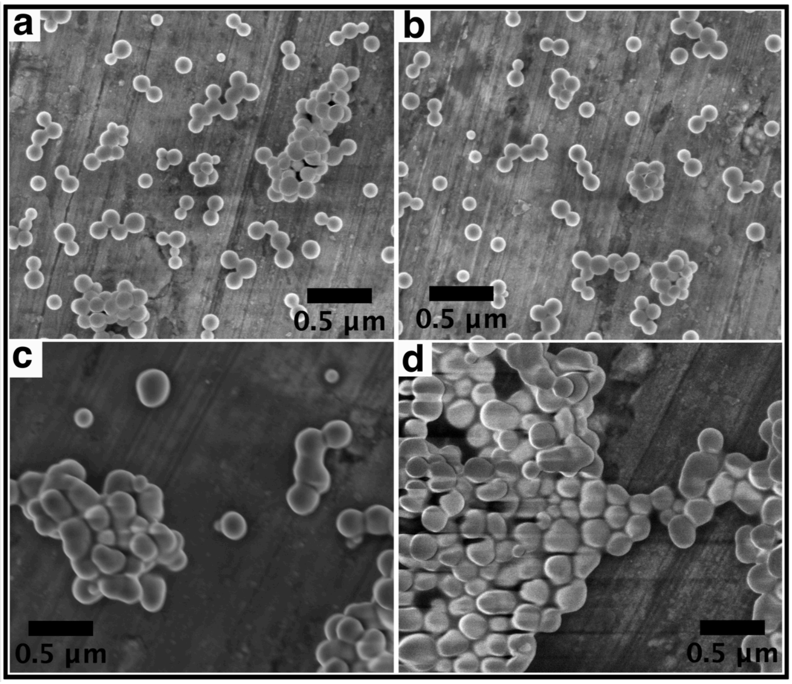

Figure S9: Low magnification SEM images obtained for **R4** in normal condition (**a** and **b**) and modifying the reactant sequence addition (**c** and **d**).

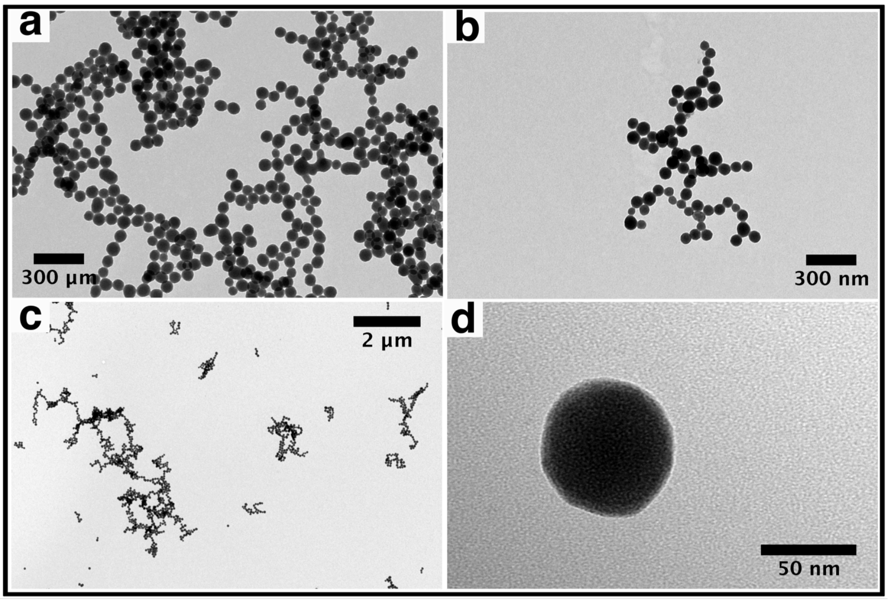

Figure S10: Low magnification electron transmission microscopy images obtained for **R2** dispersed in absolute ethanol (a and b) and in water (c and d).

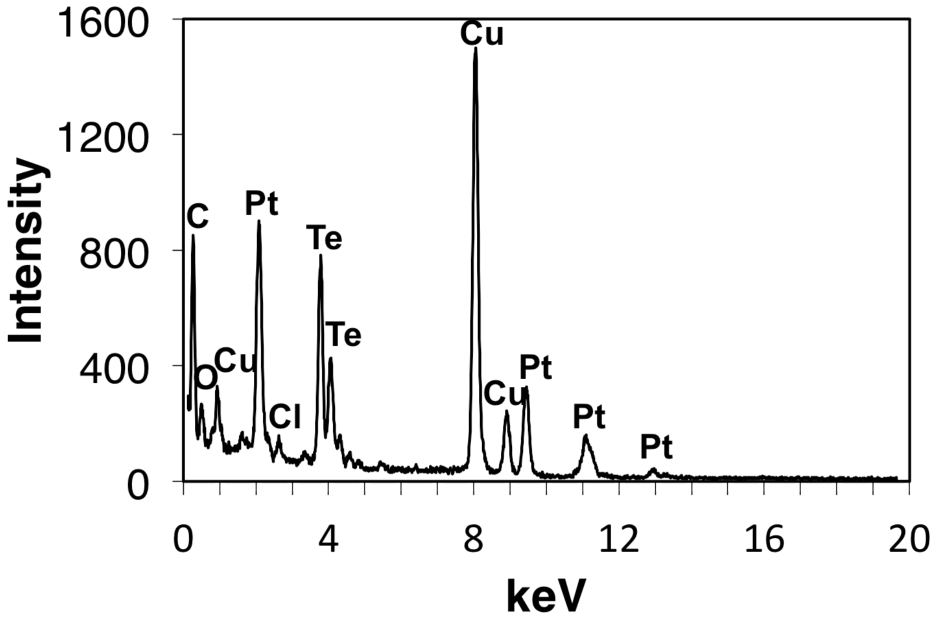

Figure S11: EDS X-ray microanalysis obtained from an individual particle

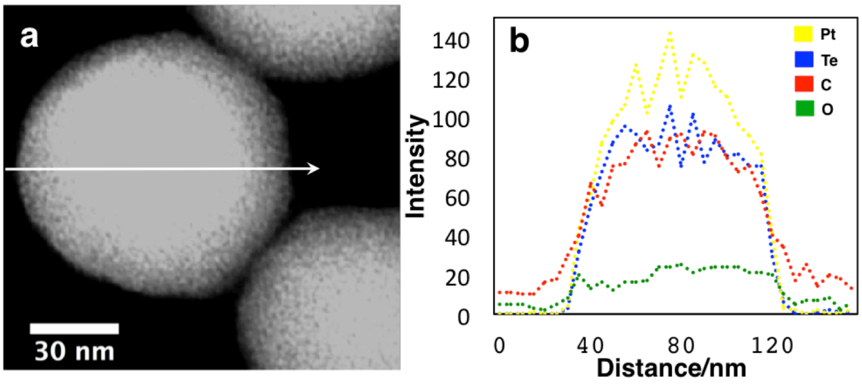

Figure S12: *(**a**) STEM-HAADF image of an as obtained organometallic Pt-Te NPs. (b) EDS intensity profiles of the PtLα_1 signal (a), and O-Kα_1, Pt-Lα_1 Te-Lα_1, and C-K$\alpha_{1\text{-}2}$ along the white arrow marked in (a).*

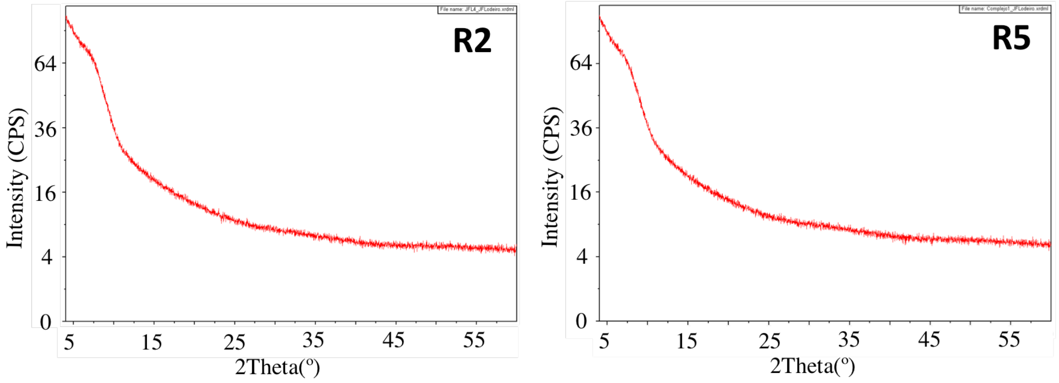

Figure S13: X-Ray diffraction pattern of organometallic Pt-Te NPs in different size.

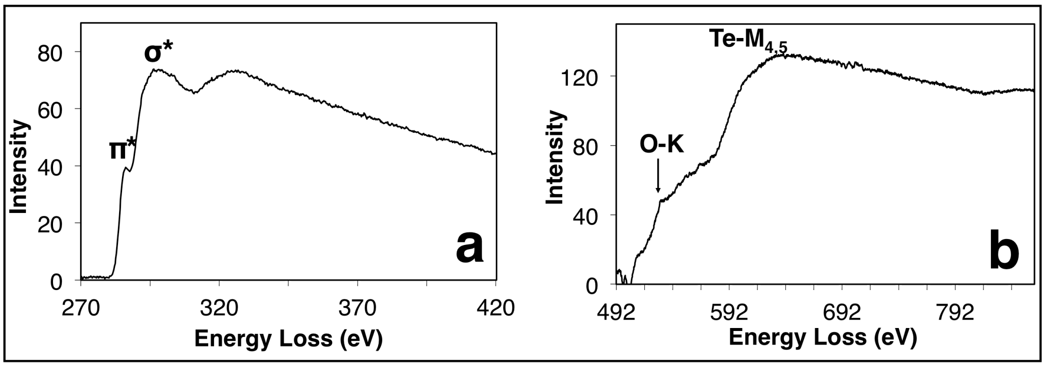

Figure S14: EEL spectrum in the region of the C K-edge (**a**) and Te, O edge(**b**) obtained for organometallic Pt-Te NPs.

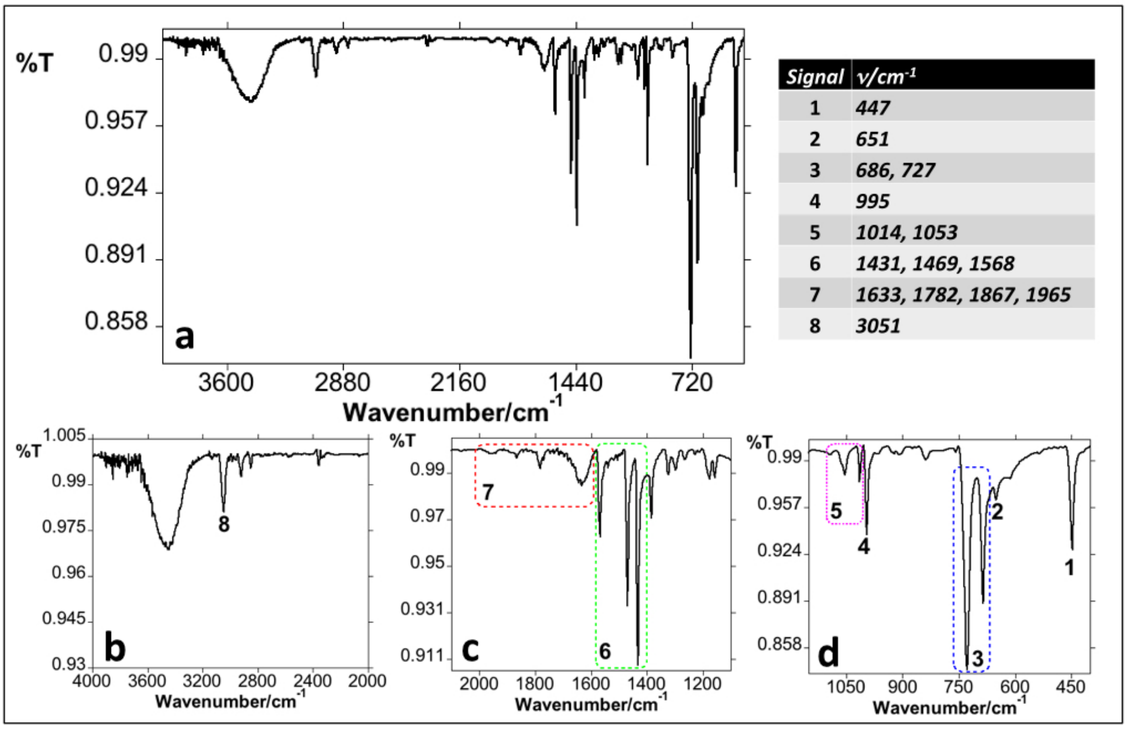

Figure S15: FT-IR spectrum (overview **a**, different spectra close-ups **b**, **c**,**d**) of organometallic Pt-Te NPs in KBr disk.

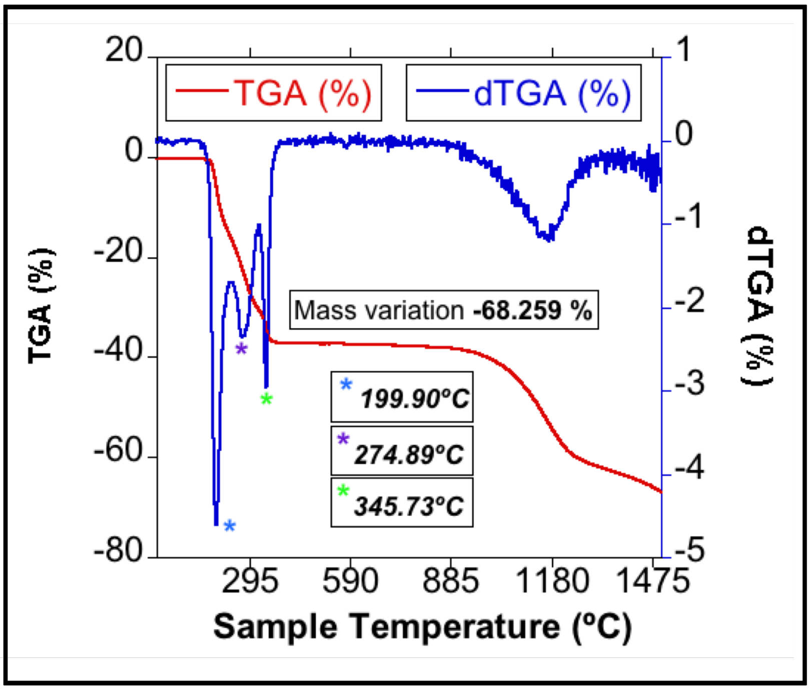

Figure S16: TG/dTG curves obtained for organometallic Pt-Te NPs.

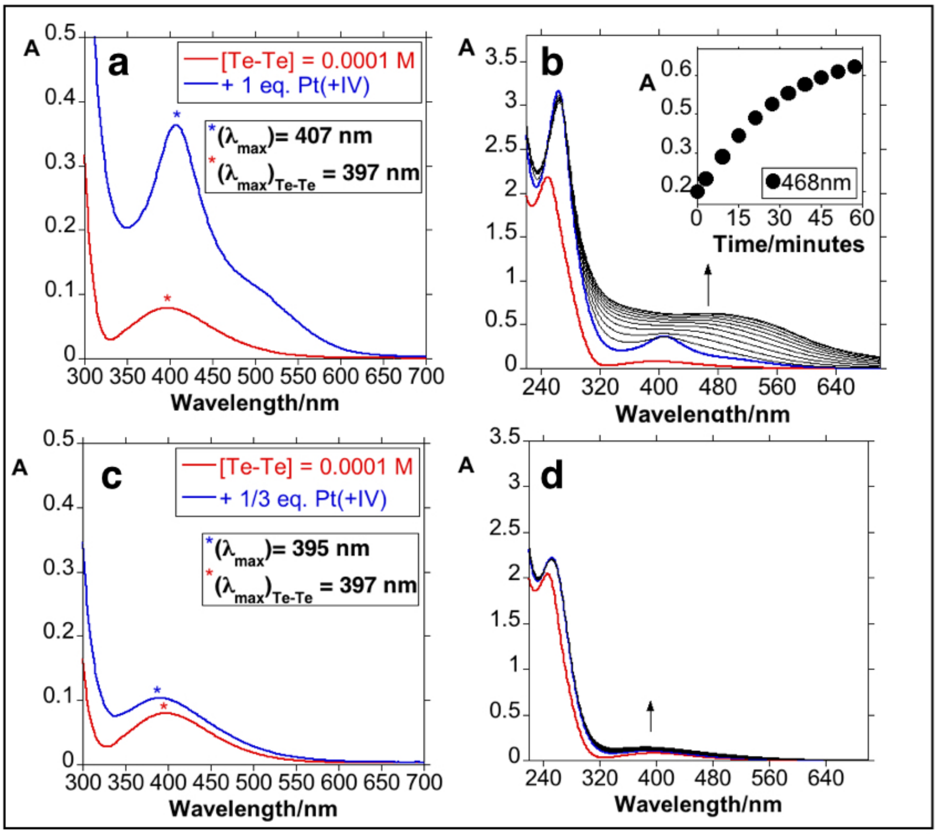

Figure S17: UV/Vis study of the time depend interaction of Ph_2Te_2 ([L]= 1.10^{-4} M) with addition of 1 (**a**, **b**) and 1/3 (**c**, **d**) equivalents of H_2PtCl_6 in acetonitrile solution.

Upon addition of 1 equivalent of Pt^{4+} a new absorption band centred in 407 nm with a shoulder at *ca.* 500 nm is observed. The evolution of the absorption spectrum over time presents an increase in the region between ca. 320 and 600 nm, with an increase in base line as derived of organometallic Pt-Te nanocomposite formation. The interaction study upon addition of 1/3 equivalent of Pt^{4+} presents a similar time dependent behaviour, but with a lower increase of absorption between 300 and 600 nm. Additionally the formation of the band centred at 407 nm is not clearly observed. A slight blue shift in Te-Te charge transfer band to 395 nm.

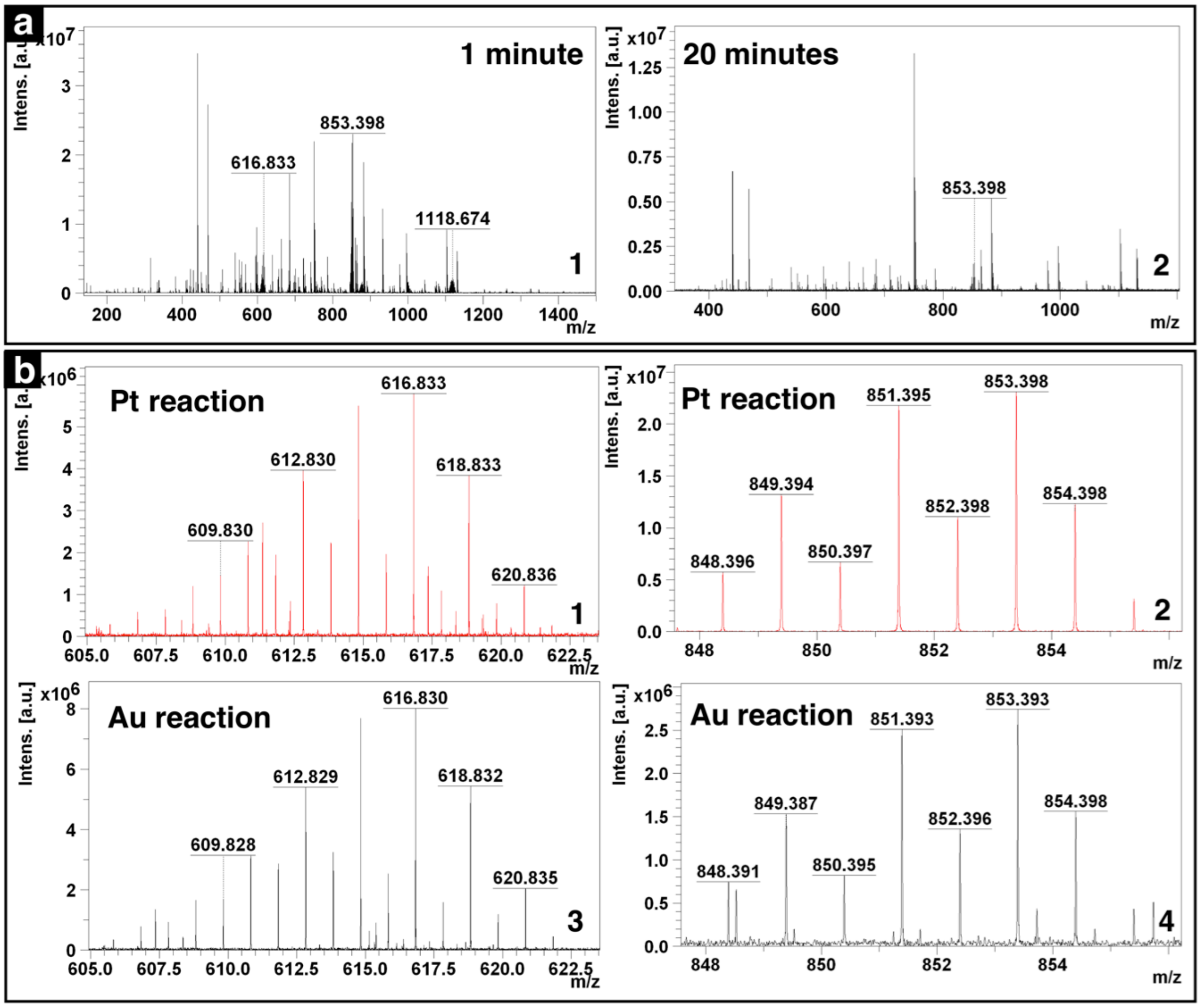

Figure S18: (**a**) FT-ICR MS (+) spectra of the reaction time at (1) 1 min, (2) 20 min. (**b**) Experimental isotopic mass spectra for the peaks at m/z 616.833 and m/z 853.398 for Pt and 616.830 m/z and 853.393 m/z for previously reported[1] gold reaction.

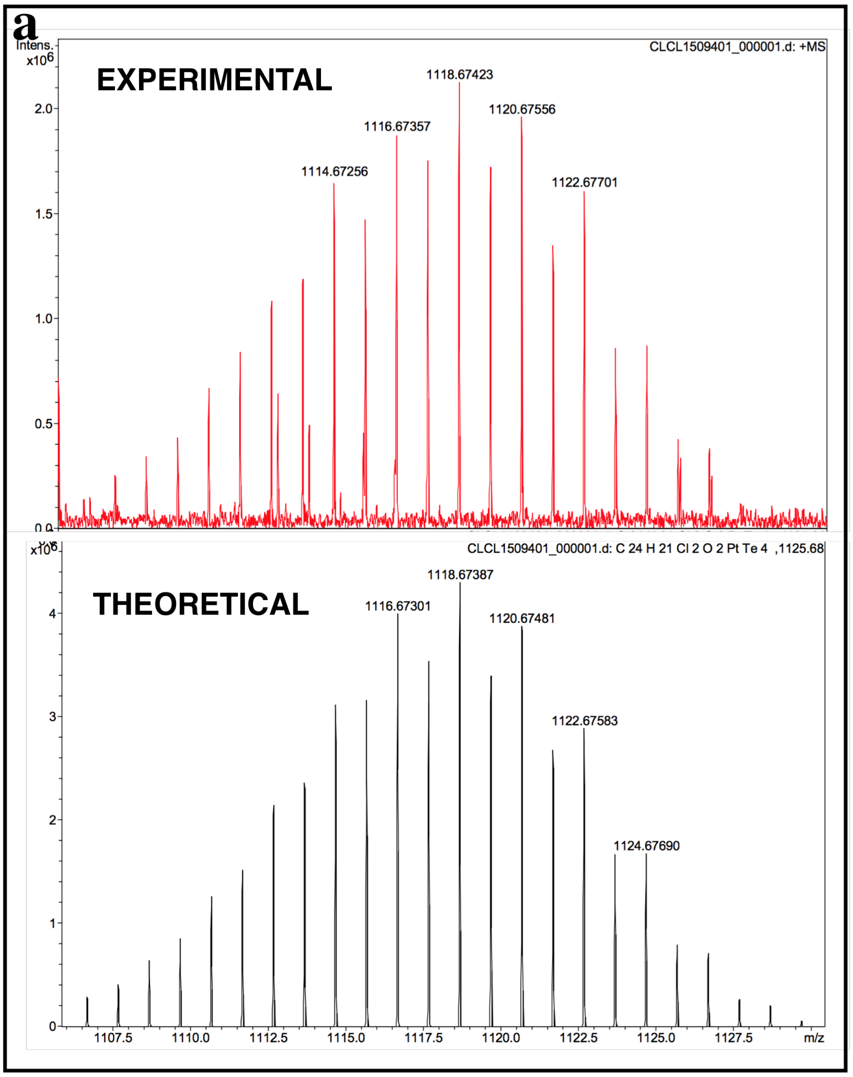

b

$[C_{24}H_{21}Cl_2O_2PtTe_4]^+$

|||

$[(PhTeO)_2.(PtCl_2).(PhTe)_2 - H]^+$

Figure S19. Experimental and theoretical isotopic pattern of 1118.674 m/z. This signal can be formed with one Pt(II) metal coordinated with one molecule of Ph_2Te_2 (blue) and one molecule of an derivative of phenyl tellurium oxidized (red) together to Cl atoms (green).

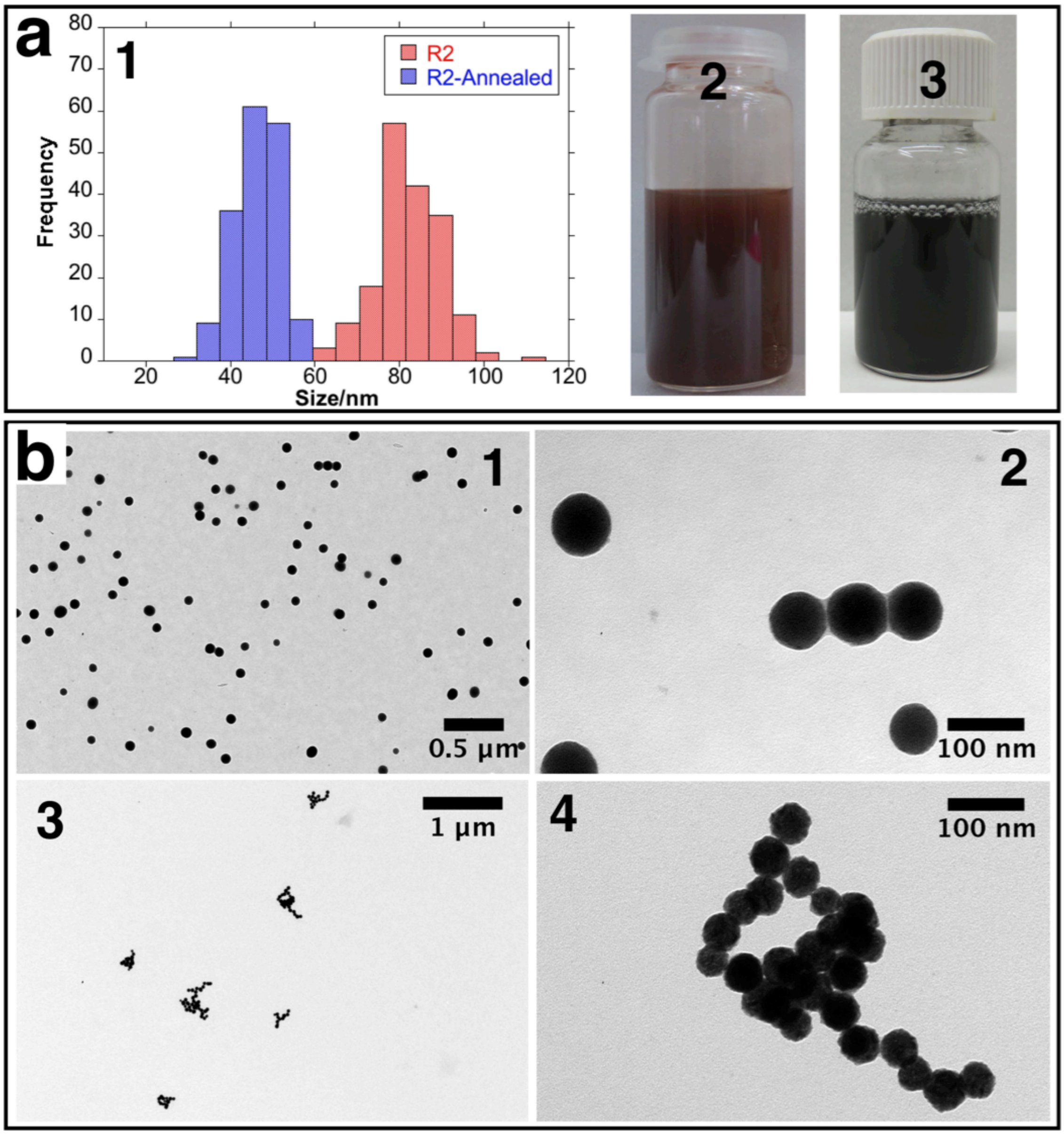

Figure S20: In panel **a** the histograms obtained for organometallic nanoparticles (sample R2) before and after the annealed process (**1**) and the colour solution of organometallic Pt-Te NPs (**2**) and annealed ($PtTe_2$ multi-crystallite) NPs (**3**) can be seen. In painel **b** images of organometallic Pt-Te NPs (1, 2) and the resulting $PtTe_2$ multi-crystallite NPs (3, 4).

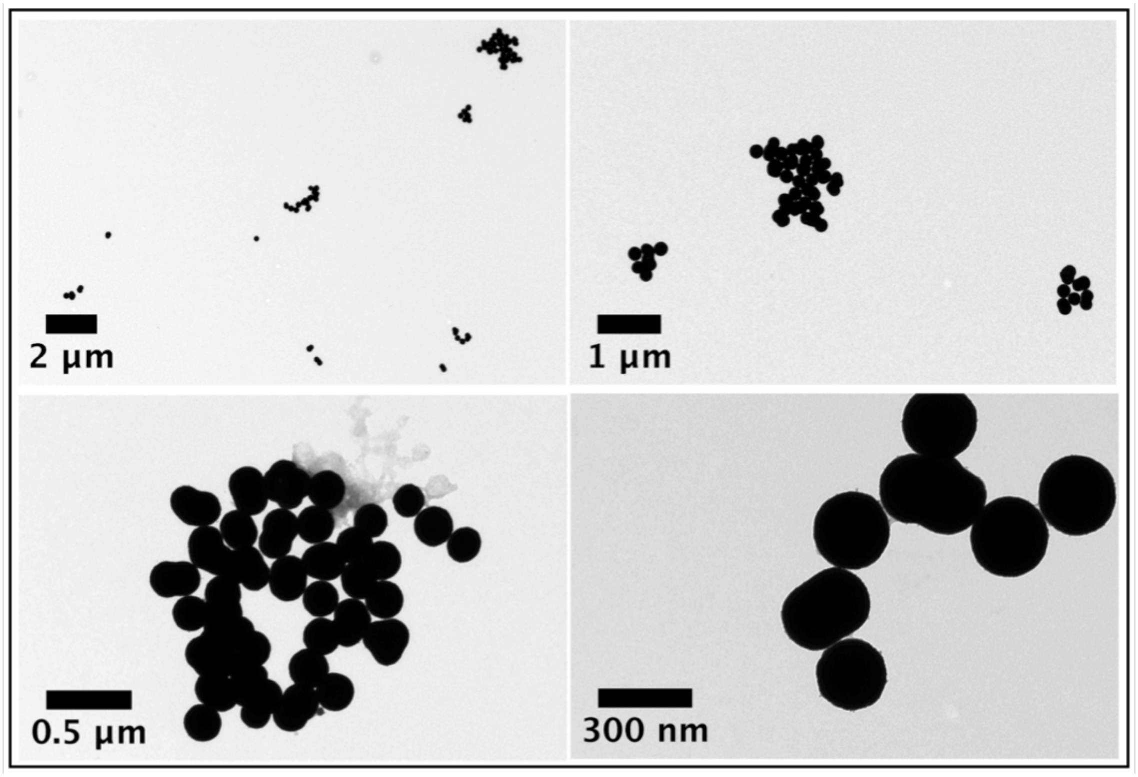

Figure S21: Low magnification electron transmission microscopy images $PtTe_2$ multi-crystallite particles obtained after annealing process of R5.

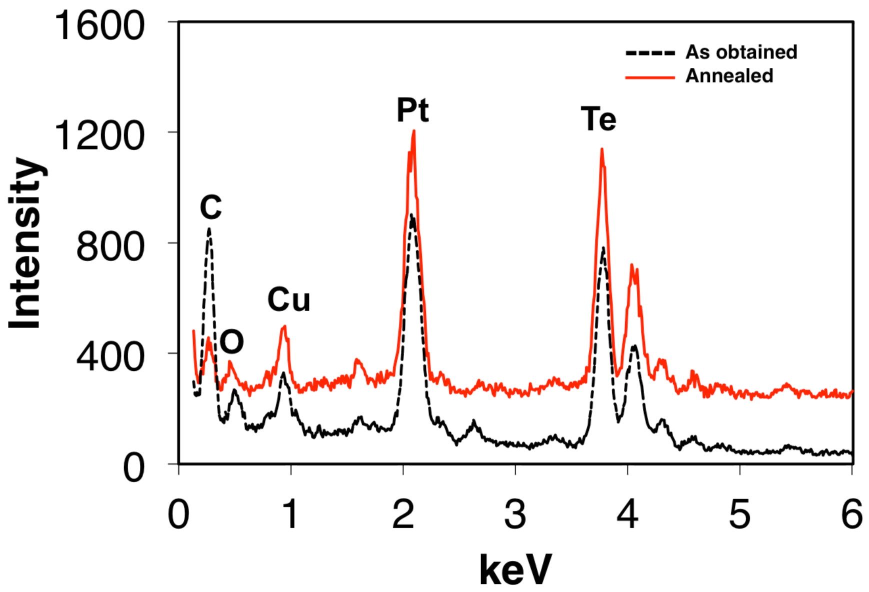

Figure S22: EDS X-ray microanalysis spectra of the as obtained organometallic Pt-Te NPs and of the annealed ($PtTe_2$ Multi-Crystallite) NPs, note the drop in the relative intensity of the C-K$\alpha_{1\text{-}2}$ signal in the annealed NPs.

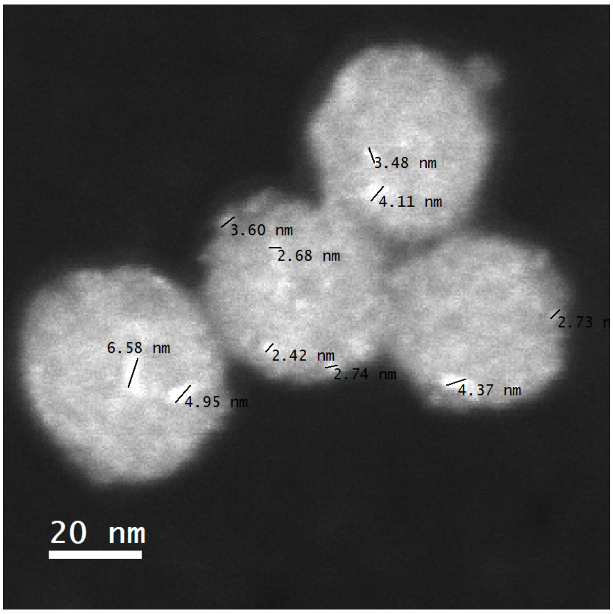

Figure S23: STEM image of a group of $PtTe_2$ multi-crystallite NPs showing crystalline contrast, brighter areas are due to the presence of small crystallites within the NPs. Numbers display the length of the black lines.

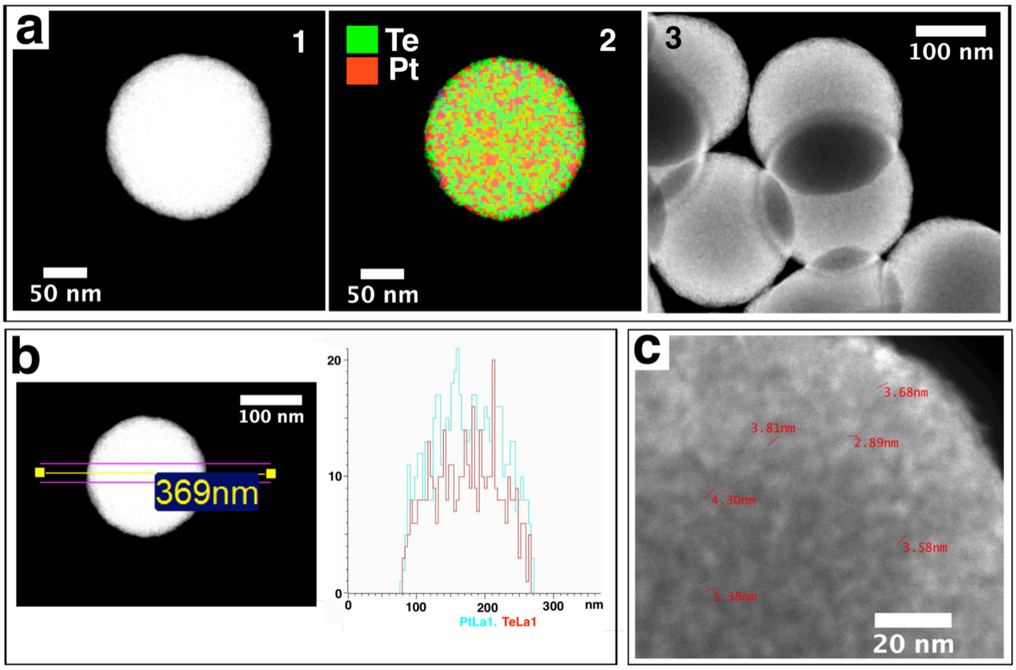

Figure S24: (**a**) STEM-HAADF image of a group isolated $PtTe_2$ multi-crystallite particles and Te, Pt EDS elemental maps (1,2) and STEM image of a group of particles. (**b**) EDS intensity profiles of the PtLα_1 and Te-Lα_1, signal along the yellow line. (**c**) STEM image in high resolution of an isolated particle showing crystalline contrast, brighter areas are due to the presence of small crystallites within the particles. Numbers display the length of the red lines.

References

(1) Fernández-Lodeiro, J.; Rodríguez-González, B.; Santos, H. M.; Bertolo, E.; Capelo, J. L.; Dos Santos, A. A.; Lodeiro, C. *ACS Omega* **2016**, *1* (6), 1314–1325.
